# Cellular Stress Responses: Cell Survival and Cell Death

**DOI:** 10.1155/2010/214074

**Published:** 2010-02-21

**Authors:** Simone Fulda, Adrienne M. Gorman, Osamu Hori, Afshin Samali

**Affiliations:** ^1^Children's Hospital, Ulm University, Eythstraße. 24, 89075 Ulm, Germany; ^2^School of Natural Sciences, National University of Ireland, Galway, University Road, Galway, Ireland; ^3^Kanazawa University Graduate School of Medical Science, Department of Neuroanatomy, Kanazawa City, Ishikawa, 920-8640 Japan, Japan

## Abstract

Cells can respond to stress in various ways ranging from the activation of survival pathways to the initiation of cell death that eventually eliminates damaged cells. Whether cells mount a protective or destructive stress response depends to a large extent on the nature and duration of the stress as well as the cell type. Also, there is often the interplay between these responses that ultimately determines the fate of the stressed cell. The mechanism by which a cell dies (i.e., apoptosis, necrosis, pyroptosis, or autophagic cell death) depends on various exogenous factors as well as the cell's ability to handle the stress to which it is exposed. The implications of cellular stress responses to human physiology and diseases are manifold and will be discussed in this review in the context of some major world health issues such as diabetes, Parkinson's disease, myocardial infarction, and cancer.

## 1. Overview of Cellular Stress Responses

Cells respond to stress in a variety of ways ranging from activation of pathways that promote survival to eliciting programmed cell death that eliminates damaged cells. The cell's initial response to a stressful stimulus is geared towards helping the cell to defend against and recover from the insult. However, if the noxious stimulus is unresolved, then cells activate death signaling pathways. The fact that the cell's survival critically depends on the ability to mount an appropriate response towards environmental or intracellular stress stimuli can explain why this reaction is highly conserved in evolution. For example, antioxidant defence mechanisms against oxidative injury and stress proteins such as heat shock proteins occur in lower organisms as well as the mammals.

There are many different types of stress and the response a cell mounts to deal with these conditions will depend on the type and level of the insult. For example, protective responses such as the heat shock response or the unfolded protein response mediate an increase in chaperone protein activity which enhances the protein folding capacity of the cell, thus counteracting the stress and promoting cell survival. The adaptive capacity of a cell ultimately determines its fate. 

Therefore, depending on the level and mode of stress, different defense mechanisms and prosurvival strategies are mounted; however, if these are unsuccessful, then the cell death programs are activated to eliminate these damaged cells from the organism. The mechanism by which a cell dies, that is, apoptosis, necrosis, pyroptosis, or autophagic cell death, often depends on its ability to cope with the conditions to which it is exposed. In this review we initially discuss the different forms of cell death that can be activated by adaptive responses because activation of death signaling pathways is the ultimate response to all types of persistent irresolvable stress. In Section 3 we will discuss the many types of stress a cell can encounter and the different responses that are activated to survive adverse conditions. Finally, we will discuss the involvement or contribution of cellular stress responses to disease states.

## 2. Stress-Induced Cell Death

Cell death has many forms and shapes. Cell death research encompasses not only the study of programmed forms of cell death (both apoptosis and autophagic cell death), necrosis and other modes of cellular demise but also the role these phenomena play in physiological and pathological processes including development, aging, and disease. 

The cell death field has attracted much attention in the last two decades, mainly because of its relevance to development, degenerative diseases, and cancer. However, the field of cell death research is by no means new [1]. The concepts of cellular demise and associated terminology have been evolving since the 19th century. The term *programmed cell death* refers to controlled or regulated forms of death associated with a series of biochemical and morphological changes [2–4]. The realization that some forms of cell death were biologically controlled or programmed has led to exploitation of these processes and has made profound impact in various fields of biology and medicine [5–7].

Nowadays, programmed cell death is synonymous with apoptosis; however, based on the original definition it also refers to autophagic cell death [8]. The term *apoptosis* was first used to describe a particular morphology of cell death [9] common to the vast majority of physiological cell deaths. This morphology includes shrinkage and blebbing of cells, rounding and fragmentation of nuclei with condensation, and margination of chromatin, shrinkage, and phagocytosis of cell fragments without accompanying inflammatory responses (in most cases) [9–11]. The morphology of cells undergoing apoptosis appeared dissimilar and distinct from the morphology associated with necrosis [9, 10]. *Necrosis*, a term commonly used by pathologists, refers to any deaths associated with the loss of control of ionic balance, uptake of water, swelling, and cellular lysis [12, 13]. This lysis releases many intracellular constituents, attracting immune cells and provoking an inflammatory response. 

### 2.1. Apoptosis

During the 1980s, apoptosis became the focus of attention, primarily because of the relative ease with which it could be distinguished morphologically from other types of cell death. Within a few years apoptosis and delineation of the underlying biochemical and molecular pathways dominated cell death research. The discoveries of the Bcl-2 family of proteins [14–16], death receptors [17], caspases [18], mitochondrial cytochrome *c *release [19], and a role for the endoplasmic reticulum [20] in apoptosis were just a few major milestones in the history of the field. Today the morphological and biochemical changes associated with apoptosis are largely explained by activation of caspases, and apoptosis has become generally accepted as caspase-dependent programmed cell death [21]. 

Of all the forms of cell death apoptosis is the best characterized and its highly regulated nature makes it an attractive target for therapeutic intervention. Apoptosis is highly conserved throughout evolution [22, 23] and plays a major physiological role in both embryonic development and aging [22, 24]. Various types of cellular stress stimuli have been shown to trigger apoptosis, including chemotherapeutic agents, irradiation, oxidative stress, and ER stress. Caspases, a family of cysteine proteases, act as common death effector molecules in various forms of apoptosis [25]. Caspases are synthesized as inactive proenzymes, which upon activation cleave various substrates in the cytoplasm or nucleus. This leads to many of the morphologic features of apoptotic cell death, for example, polynucleosomal DNA fragmentation, loss of overall cell shape, and nuclear shrinking [22, 25–27].

During apoptosis caspases are activated by different mechanisms. Stimulation of death receptors of the tumor necrosis factor (TNF) receptor superfamily such as CD95 (APO-1/Fas) or TNF-related apoptosis inducing ligand (TRAIL) receptors by their respective ligands or agonistic antibodies results in receptor aggregation and recruitment of the adaptor molecule Fas-associated death domain (FADD) and procaspase-8 to form the death inducing signaling complex (DISC) [26]. Upon recruitment caspase-8 becomes activated and initiates apoptosis by direct cleavage of downstream effector caspases [26]. The mitochondrial pathway to caspase activation is initiated by the release from the mitochondrial intermembrane space of apoptogenic factors such as cytochrome *c*, apoptosis inducing factor (AIF), second mitochondria-derived activator of caspase (Smac)/direct IAP binding protein with low pI (DIABLO) or Omi/high-temperature requirement protein A2 (HtrA2) [28]. The release of cytochrome c into the cytosol results in caspase-3 activation through formation of the cytochrome *c*/Apaf-1/caspase-9-containing apoptosome complex [29]. Smac/DIABLO or Omi/HtrA2 promotes caspase activation through neutralizing the inhibitory effects of Inhibitor of Apoptosis Proteins (IAPs) [30]. Activation of caspases has to be tightly controlled because of the potential detrimental effects on cell survival if they are inappropriately activated. For example, resistance to apoptosis can be caused by aberrant function or expression of IAPs [30]. IAPs present a group of endogenous inhibitors of caspases with eight members in human cells, that is, XIAP, cIAP1, cIAP2, survivin, livin (ML-IAP), NAIP, Bruce (apollon), and ILP-2 [30]. All IAP proteins harbor one or more baculovirus IAP repeat (BIR) domains that mediate their inhibitory interaction with caspases [30]. Among the IAP family proteins, XIAP is the most potent inhibitor of caspases and blocks apoptosis by binding to active caspase-3 and -7 and by interfering with caspase-9 activation [30]. 

In addition, the ratio of antiapoptotic versus pro-apoptotic Bcl-2 family proteins regulates apoptosis sensitivity. The Bcl-2 proteins comprise both anti-apoptotic family members, for example, Bcl-2, Bcl-X_L_, and Mcl-1, and pro-apoptotic molecules such as Bax, Bak, and BH3 domain only molecules [31]. According to the direct activation model of Bcl-2 protein activation, BH3-only proteins that function as direct activators (such as Bim and the cleaved form of Bid (tBid)), directly bind to Bax and Bak to stimulate their activation [32]. In this model, BH3-only proteins that act as sensitizers such as Bad promote apoptosis by binding to the prosurvival Bcl-2 proteins [32]. In contrast, the indirect activation model proposes that BH3-only proteins activate Bax and Bak in an indirect manner by binding to the multiple anti-apoptotic Bcl-2 proteins that inhibit Bax and Bak, which in turn leads to the release of Bax and Bak [33, 34]. Moreover, apoptosis sensitivity may be controlled by IAPs, through the regulation of additional signaling cascades, for example, the NF-*κ*B, JNK, TNFR, and the ubiquitin/proteasome pathway [30, 35]. The anti-apoptotic mechanisms regulating cell death have also been implicated in conferring drug resistance to tumor cells.

### 2.2. Autophagic Cell Death

Autophagy (self-eating) is a multistep process that is characterized by the vesicular sequestration and degradation of long-lived cytoplasmic proteins and organelles, for example, mitochondria [36]. The resulting double-membrane vesicle is termed an autophagosome [36]. The discovery of autophagy-related (*atg*) genes, first in yeast and subsequently in humans, has greatly enhanced the molecular understanding of the mechanisms that are involved in the control of autophagy [36]. The protein product of the tumor suppressor gene Beclin 1 is the mammalian homolog of Atg6 and forms a multiprotein complex together with Vps34, a class III phosphatidylinositol 3-kinase, UVRAG (UV irradiation resistance-associated tumor suppressor gene), and a myristylated kinase (Vps15, or p150 in humans) [36, 37]. This complex is required for the initiation of the formation of the autophagosome. Once this complex forms, Vps34 becomes activated and catalyzes the generation of phosphatidylinositol-3-phosphate, which is required for vesicle nucleation.

Two major protein conjugation systems exist that are required for autophagosome formation, that is, the Atg12–Atg5 conjugation and Atg8-phosphatidylethanolamine conjugation systems [38]. Mechanistically, both conjugation systems function in a manner that is closely related to ubiquitin conjugation to proteins, with corresponding conjugation-assisting enzymes that resemble the E1 and E2 enzymes in ubiquitin conjugation [38]. In the Atg12–Atg5 conjugation pathway, Atg12 is covalently conjugated to Atg5 with the help of the E1-like enzyme Atg7 and the E2-like enzyme Atg10 [36]. In the other conjugation pathway, phosphatidylethanolamine (PE) is conjugated to LC3, one of the mammalian homologues of Atg8 [36]. This process involves the sequential action of the protease Atg4, the E1-like enzyme Atg7 and the E2-like enzyme Atg3. Subsequently, lipid conjugation results in the conversion of the soluble form of LC3, that is, LC3-I, to the autophagic-vesicle-associated form that is termed LC3-II [39]. Thus, LC3 is soluble under unstressed conditions and undergoes association with peripheral membranes of autophagosomes during the induction of autophagy. Via the fusion with lysosomes, the content of autophagosomes is degraded by the action of acid-dependent enzymes [36].

Autophagy is typically observed in cells that are exposed to a variety of metabolic and therapeutic stresses, including growth factor deprivation, inhibition of the receptor tyrosine kinase/Akt/ mammalian target of rapamycin (mTOR) signaling, shortage of nutrients, ischemia/reperfusion, inhibition of proteasomal degradation, the accumulation of intracellular calcium, and endoplasmic reticulum (ER) stress [40–43]. Reactive oxygen species (ROS) may provide a common link between cellular stress signals and the initiation of autophagy, as ROS accumulation has been reported to result in inactivation of the cysteine protease ATG4, which in turn causes accumulation of the ATG8-phosphoethanolamine precursor that is required for the initiation of autophagosome formation [44]. The functional relationship between autophagy and cell death is complex in the sense that, under most cellular settings, autophagy functions as a stress adaptation that prevents cell death, whereas in some circumstances, it constitutes an alternative route to cell death. This complex interrelationship between autophagy and cell death implies that these responses are somewhat linked at the molecular level. However, the key molecular events that eventually determine whether autophagy is protective or destructive are still poorly understood.

Although it is still controversial whether autophagy is protective or toxic for the cells, accumulating evidence suggests that it has beneficial roles in the heart under both physiological and pathological conditions [45, 46]. Autophagy was shown to mediate turnover of intracellular proteins and organelles in the heart and protect against hemodynamic stress [45]. Consistent with this, rapamycin, which induces autophagy by inhibiting mTOR, can protect myocardium against ischemia/reperfusion injury [47]. In contrast, recent studies also demonstrated that downregulation of the transcription factors, activating transcription factor 5 or 7 (ATF5 or ATF7), using siRNA prevented stress-induced cell death [48, 49], suggesting that the level or timing of autophagy may be critical for deciding the fate of the cells. Autophagic cell death has mainly been shown during development. However, during recent years accumulating evidences suggest that inhibition of apoptosis induces cell death that is either associated with or dependent on autophagy [48–50]. There is evidence of cross-talk between apoptosis and autophagy at the molecular level, particularly with regard to the Bcl-2 family. In addition to its role in inhibiting apoptosis, Bcl-2 has also been shown to inhibit autophagy [51, 52] and autophagic cell death [53]. This effect is mediated through the ability of Bcl-2 to interact with Beclin 1, a key protein in autophagosome formation [52]. In fact, Beclin 1 has been shown to be a novel BH3-only protein and to interact with a number of anti-apoptotic Bcl-2 family members including Bcl-2, Bcl-xL, Bcl-w, and Mcl-1 [54–57].

### 2.3. Necrosis

Necrosis has been considered as an accidental mode of cell death for many years, implying that within a multicellular organism it is an unregulated process. However, there is now mounting evidence that the execution of necrotic cell death is also regulated by a set of signaling pathways [58–60]. For instance, death domain receptors, for example, TNFR1, and Toll-like receptors have been reported to trigger necrosis, in particular in the presence of caspase inhibitors [58]. In addition, necrotic cell death has been reported in response to cellular stress stimuli, including ischemia or glutamate excitotoxicity in neurons or cancer cells exposed to alkylating DNA damaging agents [61–63]. Morphologically, necrosis is characterized by a gain in cell volume, swelling of organelles and plasma membrane rupture, which results in the loss of intracellular contents. Several signal transduction cascades have been described that are involved in the propagation of necrotic cell death. There is mounting evidence that the serine/threonine kinase RIP1 is one of the key mediators of necrotic cell death, at least in the case of death receptors or Toll-like receptors [64, 65]. Studies in RIP1-deficient leukemia cells revealed that RIP1 is required for death receptor-induced necrosis [66, 67]. Furthermore, RIP1 has been described to be required for lipopolysaccharide-induced cell death of macrophages [68]. In line with a central role of RIP1 in necrotic cell death, small molecule inhibitors of RIP1 kinase were reported to protect against ischaemic brain injury in an in vivo model of necrosis [69–71]. In addition to RIP1, there is very recent evidence that RIP3 is also critical for necrotic cell death [72–74]. To this end, RIP3 was identified in an RNA interference screen to be essential for necrosis in response to TNF*α* stimulation and during virus infection [72, 73]. RIP3 interacts with RIP1 and regulates RIP1 phosphorylation and the generation of ROS [72–74].

Moreover, ROS and calcium constitute important mediators that are involved in the propagation of the necrotic signal in various forms of necrosis, for example, upon stimulation with TNF*α* or exposure to double-stranded DNA [75, 76]. ROS may be generated intracellularly by mitochondria and glycolysis [75, 77]. While the ER is the main intracellular calcium store, mitochondrial calcium has been described to stimulate oxidative phosphorylation, thereby promoting ROS generation [78]. Both ROS and calcium can cause damage to organelles and macromolecules, which contributes to the loss of cell integrity. In addition calcium-mediated activation of calpain can lead to cleavage and inactivation of caspases [79], whereas the ROS can target the active site of caspases and render them inactive [80]. Many stimuli that drive necrosis can inhibit the apoptotic machinery.

## 3. Cellular Stress Responses

During tissue homeostasis there is an equilibrium between the net growth rate and the net rate of cell death [22]. Upon exposure to cellular stress this physiological homeostasis is in danger. Depending on the type of cellular stress and its severity, the cell's response can be manifold. In essence, if the stress stimulus does not go beyond a certain threshold, the cell can cope with it by mounting an appropriate protective cellular response, which ensures the cell's survival. Conversely, the failure to activate or maintain a protective response, for example, if the stressful agent is too strong, results in activation of stress signaling cascades that eventually fuel into cell death pathways [81, 82]. 

### 3.1. The Heat Shock Response

One of the main prosurvival activities of cells, the heat shock response, was originally described as the biochemical response of cells to mild heat stress (i.e., elevations in temperature of 3–5°C above normal) [83, 84]. It has since been recognized that many stimuli can activate this response, including oxidative stress and heavy metals. One of the main cellular consequences of these stresses is protein damage leading to the aggregation of unfolded proteins. In order to counteract this, cells increase the expression of chaperone proteins that help in the refolding of misfolded proteins and alleviate protein aggregation. This confers a transient protection, leading to a state that is known as thermotolerance, whereby cells become more resistant to various toxic insults, including otherwise lethal temperature elevations, oxidative stress, various anticancer drugs, and trophic factor withdrawal [85–88].

During initiation of the heat shock response general protein transcription and translation is halted, presumably to alleviate the burden of misfolded proteins in the cell. However, transcription factors that enhance expression of a specific subset of protective genes are selectively activated under these conditions; these are the heat shock factors (HSFs) [89]. Vertebrate cells have three different HSFs: HSF1 is essential for the heat shock response and is also required for developmental processes, HSF2 and HSF4 are important for differentiation and development, while HSF3 is only found in avian cells and is probably redundant with HSF1 [90, 91]. Cells derived from mice lacking HSF1 are sensitive to stress and are unable to develop thermotolerance or induce heat responsive genes upon heat shock [92–94], which has confirmed that HSF1 in particular is responsible for the heat shock response. More recent work has shown that HSF2 can modulate HSF1-mediated expression of heat-responsive genes [95], suggesting that HSF2 also participates in transcriptional regulation of the heat shock response.

**Figure 1:**
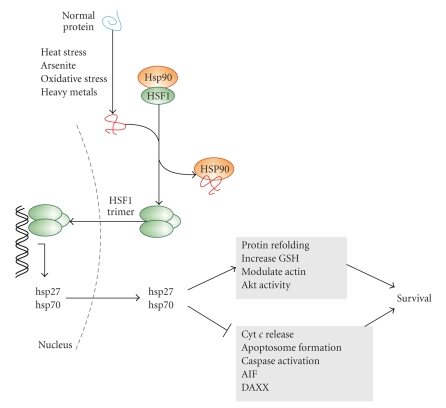
Induction of heat shock proteins inhibits apoptosis and promotes cell survival. Exposure of cells to elevated temperatures, oxidative stress, and heavy metals causes accumulation of unfolded proteins, which through activation of HSF1 leads to induction of Hsp27 and Hsp70. These Hsps inhibit apoptosis and promote survival.

Inactive HSF1 is maintained in a monomeric form in the cytoplasm through interaction with Hsp90 and cochaperones [96, 97] (Figure 1). When the cell is exposed to stressful conditions, there is accumulation of unfolded proteins which compete with HSF1 for Hsp90 binding. Thus, HSF1 is released from the complex stimulating its transition from a monomer to a homotrimer that can translocate to the nucleus and bind to DNA (Figure 1). HSFs bind to upstream sequences (heat shock elements) in the promoters of target genes, leading to the expression of heat shock proteins (Hsps). 

Hsps are a set of evolutionary conserved proteins that are grouped into subfamilies with molecular weights of approximately 110, 90, 70, 60, 40, and 15–30 kDa [85, 98]. Some of these, for example, Hsp90, are constitutively expressed and act intracellularly as molecular chaperones, preventing premature folding of nascent polypeptides [99]. Others, particularly Hsp27 and Hsp70, are usually expressed at low basal levels and increase in response to environmental and physiological stressors, and as such they are termed inducible Hsps and are part of the heat shock response [85]. Hsp27 belongs to a subfamily of stress proteins, the small Hsps, which are detectable in virtually all organisms. Hsp27 is also regulated by phosphorylation and dynamic association/dissociation into multimers ranging from dimers to large oligomers [100]. Hsp70 is the inducible member of the 70 kDa family of Hsps. Both Hsp27 and Hsp70 have been shown to protect cells against the induction of cell death by a variety of stresses and by different modes of cell death, including apoptosis [86, 101] and necrosis [102–104]. They achieve these effects directly, through inhibition of cell death pathways, and indirectly, through general prosurvival activities. For example, in their capacity as molecular chaperones, inducible Hsps bind to and aid the refolding of unfolded proteins, thereby preventing protein aggregation [105]. Hsp27 can interact with actin and is thus important for maintaining the integrity of the cytoskeleton which may play a role in promoting survival [106].

Apart from these indirect mechanisms, Hsp27 and Hsp70 can directly inhibit apoptosis by modulating both the intrinsic and the extrinsic apoptosis pathways and by interfering with caspase activation at several different levels [107–109]. Both Hsp27 and Hsp70 have been reported to directly block release of pro-apoptotic factors, including cytochrome *c*, from the mitochondria [110–112]. In the cytosol, these Hsps can block apoptosome formation and activation of downstream caspases through their ability to bind to cytochrome *c* and procaspase-3 (in the case of Hsp27) [107, 108] and procaspases -3, -7 and Apaf-1 (in the case of Hsp70) [101, 113–115]. Hsp70 can also interact with and inhibit apoptosis-inducing factor (AIF) thus inhibiting apoptotic nuclear changes [116, 117]. Hsps can also modulate the death receptor pathway. Hsp27 is reported to inhibit DAXX, an adaptor protein that links the Fas death receptor and the ER stress sensor IRE1 to ASK-1 and downstream JNK pro-apoptotic signaling [118]. Hsp70 also inhibits JNK activity [119–121], although this is not observed in all systems [101]. Hsp27 and 70 can also interact with other proteins that regulate cell survival. For example, Hsp27 can interact with the prosurvival Ser/Thr kinase Akt which is suggested to be important for sustained Akt activity [122–124]. Hsp70 can exist in complex with cochaperones, including DnaJ/Hsp40 and BAG-1 which affect its ability to modulate apoptosis [125, 126]. Overall, Hsps can be activated or induced by a number of stresses and they act to protect the cell by influencing a variety of cellular processes which determine cellular fate. Hsps are, in general, prosurvival and anti-apoptotic molecules.

### 3.2. The Unfolded Protein Response (UPR)

**Figure 2:**
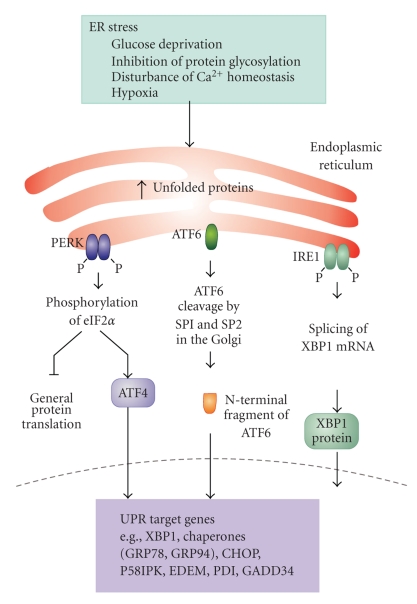
*ER stress and the unfolded protein response.* Stress to the ER stimulates the activation of the three endoplasmic reticulum (ER) stress receptors, PKR-like ER kinase (PERK), activating transcription factor 6 (ATF6) and inositol-requiring enzyme 1 (Ire1) that are involved in the unfolded protein response (UPR). PERK phosphorylates eukaryotic initiation factor 2 (eIF2*α*) which inhibits general protein translation, allowing eIF2*α*-independent translation of ATF4, which activates transcription of chaperones such as GRP78. ATF6 undergoes specific proteolysis in the Golgi apparatus which leads to activation. One of the ATF6 target genes is XBP1. IRE1 catalyzes the alternative splicing of XBP1 mRNA leading to expression of the active XBP1 transcription factor. Together the three arms of the UPR block protein translation, increase chaperone expression and enhance ER-associated protein degradative pathways.

Secretory and membrane proteins undergo posttranslational processing, including glycosylation, disulfide bond formation, correct folding, and oligomerization, in the ER. In order to effectively produce and secrete mature proteins, cellular mechanisms for monitoring the ER environment are essential. Exposure of cells to conditions such as glucose starvation, inhibition of protein glycosylation, disturbance of Ca^2+^ homeostasis and oxygen deprivation causes accumulation of unfolded proteins in the ER (ER stress) and results in the activation of a well orchestrated set of pathways during a phenomenon known as the unfolded protein response (UPR) [127, 128] (Figure 2). The UPR is generally transmitted through activation of ER resident proteins, most notably inositol-requiring protein-1 (IRE1), protein kinase RNA (PKR)-like ER kinase (PERK), and activating transcription factor 6 (ATF6). In some cells/tissues, additional ATF6-like bZip type transcription factors such as OASIS, CREB-H, Tisp40, and Luman also transmit the UPR signaling [129–132]. The UPR target genes include molecular chaperones in the ER, folding catalysts, subunits of translocation machinery (Sec61 complex), ER-associated degradation (ERAD) molecules and anti-oxidant genes [127]. 

Among the UPR transmitters so far identified, IRE1 and PERK are both type I transmembrane protein kinases which dimerize to promote autophosphorylation and activation in response to ER stress. Activated IRE1 endonucleolytically cleaves mRNA that encodes a transcription factor named homologous to ATF/CREB1 (Hac1) in yeast [133, 134] and X-box binding protein-1 (XBP1) in higher species [135, 136]. The spliced forms of Hac1 and/or XBP1 in turn activate the transcription of the UPR target genes. In contrast, activated PERK phosphorylates the *α*-subunit of eukaryotic translation initiation factor-2 (eIF2*α*) which leads to lower levels of eIF2 and translational suppression [137]. The PERK-eIF2*α* signaling pathway also activates the transcription of the UPR target genes through CAP-independent upregulation of the translation of a transcription factor ATF4 [138]. PERK can also directly phosphorylate and activate the transcription factor, NF-E2-related factor-2 (Nrf2), which contributes to cellular redox homeostatis by inducing the expression of anti-oxidant genes [139, 140]. ATF6 is a type II transmembrane protein which is cleaved by Golgi apparatus-resident proteases site-1 protease (SP1) and site-2 protease (SP-2) in response to ER stress [141, 142]. The cleaved N-terminal fragment of ATF6 acts as a transcription factor to increase the transcription of the UPR target genes together with XBP1 and ATF4. 

UPR signaling generally promotes cell survival by improving the balance between the protein load and the folding capacity in the ER and/or by improving the secretion of trophic factors/growth factors [143, 144]. However, if the protein load in the ER exceeds its folding capacity, or some defects in the UPR exist, cells tend to die, typically, with apoptotic features (ER stress-induced cell death). Although the exact molecular mechanisms that regulate this type of cell death remain to be elucidated, at least three pathways have been identified as being involved: the caspase-12/caspase-4 pathway and CHOP and IRE1-JNK pathways. Caspase-12 [145] in mice and caspase-4 in human [146] have been proposed as caspases that initiate ER stress-induced cell death. Caspase-12 null mice are reported to be relatively resistant to ER stress and amyloid-beta toxicity [145]. Caspase-12 is reported to directly cleave procaspase-9 without involvement of the cytochrome *c*/Apaf-1 pathway [147]. C/EBP homologous protein (CHOP), a transcription factor that is induced downstream of PERK and ATF6 pathways, induces ER stress-induced cell death at least in part by suppressing the expression of Bcl-2 [148] and inducing Bim expression [149]. IRE1 also participates in ER stress-induced cell death by activating JNK through the binding with ASK1 and Traf2 [150, 151].

Important roles for ER stress and ER stress-induced cell death have also been demonstrated in a broad spectrum of pathophysiological situations, including ischemia, diabetes, atherosclerosis, endocrine defects, development, neurodegenerative disorders, and cancer as described below [143, 144, 152–155].

Among the UPR targets, glucose-regulated proteins (GRPs) are the most studied and best characterized. GRPs were originally identified as proteins induced by glucose starvation [156]. Later, it was found that these molecules were transcriptionally induced by ER stress through the *cis*-acting element termed ER stress response element (ERSE) [157]. GRPs include molecular chaperones in the ER such as GRP78/Bip, GRP94, ORP150/GRP170, and oxidoreductases in the ER such as PDI, ERp72, and GRP58/ERp57. Accumulating evidence suggests that GRPs promote cell survival when exposed to stresses such as hypoxia/ischemia [143, 158], glutamate excitotoxicity [159], and neurodegeneration [160–162]. GRP78 could be a potential factor to inhibit atherosclerosis by preventing ER stress-induced cell death in endothelial cells [163]. This involves the inhibition of the activation of SREBPs, a molecule that induces cholesterol and triglyceride biosynthesis, or by inhibiting tissue factor procoagulant activity [164–166]. ORP150/GRP170 was found to be associated with insulin sensitivity in both human and mice as described below. Furthermore, GRPs also play important roles in survival during early mammalian development [159, 167–169]. 

Interestingly, recent studies have revealed that small compounds that mimic the functions of GRPs (chemical chaperones) and those that induce endogenous GRPs (molecular chaperone inducers) can prevent protein aggregation [170], improve protein secretion [171], and protect cells against brain ischemia [172] or neurodegeneration [173]. These results suggest that the regulation of ER stress can be a novel therapeutic target in a variety of diseases.

### 3.3. The DNA Damage Response

**Figure 3:**
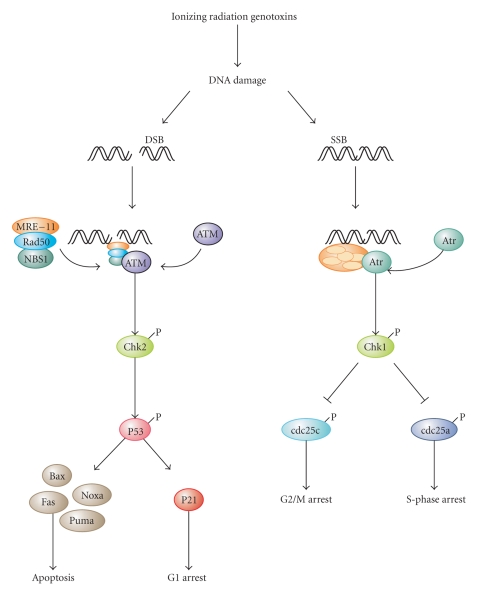
*DNA damage responses and cell death.* Upon exposure to ionizing radiation or genotoxins, the damage to DNA is a common initial event. DNA double strand breaks (DSBs) or single strand breaks (SSBs) are considered to be key lesions that initiate activation of the DNA damage response. Upon DSBs, ataxia telangiectasia mutated (ATM) is recruited by the MRE-11-Rad50-NBS1 (MRN) complex to sites of broken DNA and phosphorylates downstream substrates such as checkpoint kinase 2 (Chk2), which subsequently phosphorylates p53. Sublethal damage to DNA can engage survival pathways via p21-mediated cell cycle arrest. Alternatively—if the damage is too severe to be repaired—pro-apoptotic p53 target genes are activated including Bax, Puma, Noxa, and Fas, which promote apoptosis. Upon SSBs, it is ataxia telangiectasia and Rad3 related (ATR) that gets activated and phosphorylates Chk1. Chk1 in turn phosphorylates and inhibits cdc25c to mediated G2/M arrest or alternatively cdc25a to promote S-phase arrest.

Upon cellular stress conditions that are caused by exposure to chemotherapeutic agents, irradiation, or environmental genotoxic agents such as polycyclic hydrocarbons or ultraviolet (UV) light, damage to DNA is a common initial event [174, 175]. DNA double strand breaks (DSBs) and single strand breaks (SSBs) are considered as key lesions that initiate the activation of the DNA damage response [174]. Since the DNA duplex is more vulnerable to chemical attack or nucleases when it is separated into two single-stranded DNA strands, for example, during DNA replication and transcription, SSBs are preferentially generated under these conditions [176]. Defined SSBs are also generated during distinct pathways of DNA repair, for example, in the course of nucleotide excision repair (NER). After DNA damage recognition, dual incision 5′ to the DNA lesion by ERCC1-XPF and 3′ to the damage by XPG results in the removal of the lesion-containing oligonucleotide [177]. DSBs are produced directly or indirectly by many anticancer drugs, including DNA intercalating, alkylating or crosslinking agents, topoisomerase inhibitors, and nucleotide analogs [174]. Once DSBs are generated, ataxia telangiectasia mutated (ATM) is recruited by the MRE-11-Rad50-NBS1 (MRN) complex to sites of broken DNA and phosphorylates downstream substrates such as checkpoint kinase 2 (Chk2) and p53 [175, 178] (Figure 3). p53 induces transcriptional activation of different functional programs, for example, cell cycle regulatory proteins such as p21 and pro-apoptotic factors such as CD95, PUMA, and BAX [179]. In addition, recent studies have also defined a nontranscriptional pro-apoptotic activity of p53 that regulates the intrinsic mitochondria-mediated pathway of apoptosis [180]. Damage to DNA engages DNA repair processes to ensure the cell's survival in the case of sublethal damage [174]. Alternatively, if the damage is too severe to be repaired—the DNA-damaging insult is transmitted by the cellular stress response to the activation of effector systems to mediate cell death [174]. In the latter case, various stress-inducible molecules, including NF-*κ*B, p53, JNK, or MAPK/ERK, have been implicated in propagating and modulating the cell death signal [81, 82].

Depending on the type of lesion, DNA damage initiates one of several mammalian DNA repair pathways, which eventually restore the continuity of the DNA double strand. There are two main pathways for the repair of DSBs, that is, nonhomologous end-joining and homologous recombination [181, 182]. The former constitutes the predominant DNA repair pathway in humans and involves DNA repair proteins such as DNA-PK, Ku70, and Ku80 [181, 182]. Base damage can be repaired either by enzyme-catalyzed reversal or alternatively via excision repair [183]. Mismatch repair is responsible for the removal of incorrectly paired nucleotides [184]. It is important to note that DNA repair can, in principle, be error-free and error-prone. Several proteins have been discovered recently that exert a specific function in error-free repair processes to guarantee high-fidelity reconstitution of the DNA [185]. Faithful genome transmission requires the coordination of this highly complex network of DNA repair pathways and repair surveillance mechanisms linked to cell cycle checkpoints as well as cell death mechanisms [185]. Error-prone repair or complete failure of DNA repair cannot only lead to mutations but can also lead to the initiation of cell death pathways [185].

### 3.4. The Response to Oxidative Stress

**Figure 4:**
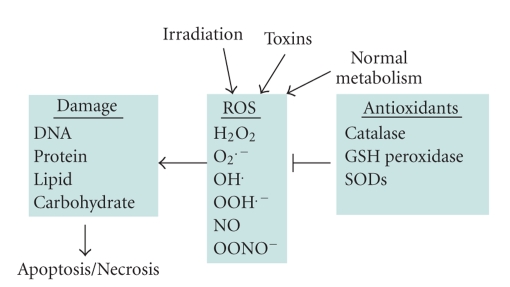
*Oxidative stress and cell death.* There is a plethora of stimuli that can trigger the generation of reactive oxygen species (ROS), among them irradiation, toxins, and also normal metabolic processes. A range of different ROS species have been identified, which are kept in check by antioxidant defenses. These include several detoxifying enzymes, for example, catalase, GSH peroxidase, and superoxide dismutase (SOD). If these antioxidants defense mechanisms are too weak, ROS-mediated damage to cellular macromolecules will eventually lead to cell death.

Cell survival requires appropriate proportions of molecular oxygen and various antioxidants. Reactive products of oxygen are amongst the most potent and omnipresent threats faced by cells. These include ROS such as superoxide anion (O_2_^•−^), hydrogen peroxide (H_2_O_2_), singlet oxygen, hydroxyl radical (OH^•^), peroxy radical, as well as the second messenger nitric oxide (NO^•^) which can react with O_2_^•−^ to form peroxynitrite (ONOO^−^). Normally in cells there exists equilibrium between pro-oxidant species and antioxidant defense mechanisms such as ROS-metabolizing enzymes including catalase, glutathione peroxidase, and superoxide dismutases (SODs) and other antioxidant proteins such as glutathione (GSH) (Figure 4). Oxidative stress occurs when there is a disturbance in this pro-oxidant:antioxidant balance and it has been implicated in several biological and pathological processes [186]. Although most oxidative insults can be overcome by the cell's natural defenses, sustained perturbation of this balance may result in either apoptotic or necrotic cell death [186–190]. 

ROS can emanate from intracellular or extracellular sources. Auto-oxidation of reduced respiratory components of the mitochondrial electron transport chain causes the production of free radical intermediates, O_2_^•−^ and H_2_O_2_, which in the presence of iron can produce highly reactive OH^•^ radical via the Fenton reaction. These ROSs are dealt with by SODs, enzymes considered to be the first line of defense against oxygen toxicity. ROS can also be produced in the cytosol. For example, the arachidonic acid cascade, yielding prostaglandins, and leukotrienes may generate ROS when the released lipid is metabolized [191], and some cytochrome P-450 isozymes are well-known ROS producers [192]. Also, the auto-oxidation reactions of ascorbic acid, low molecular weight thiols, adrenalin, and flavin coenzymes can cause ROS production. In many of these cases, cytosolic GSH neutralizes the offenders. In addition to physiological sources of ROS, diverse exogenous agents can contribute to the intracellular production of free radicals. Most of these compounds cause the generation of O_2_^•−^ and H_2_O_2_ [80, 193, 194]. The mechanism of action of many exogenous agents involves redox cycling whereby an electron is accepted to form a free radical and it is then transferred to oxygen.

Interestingly, there is evidence of cross-talk between oxidative stress and other stress response pathways. For example, oxidative stress is known to cause an increase in the expression of certain inducible Hsps, particularly Hsp27 [195–197]. Hsps have been reported to protect against many stresses apart from heat shock, including heavy metals, radiation, nitric oxide, and other oxidants. In addition, activation of the UPR stimulates upregulation of antioxidant genes through PERK-dependent phosphorylation of the Nrf2 transcription factor, whose target genes include enzymes involved in GSH biosynthesis, and heme oxygenase-1 [198]. Moreover, perturbations in cellular redox status sensitize cells to the harmful effects of ER stress [199]. Similarly, accumulating evidence suggests a role for O_2_^•−^ in the activation of autophagy [200]. 

ROS can cause damage to all of the major classes of biological macromolecules, including nucleic acids, proteins, carbohydrates, and lipids. When the cell's antioxidant defenses are overwhelmed, ROS can induce cell death. Numerous, recent studies have shown that the mode of cell death that occurs depends on the severity of the insult [187–189]. In fact, oxidants and antioxidants not only determine cell fate, but can also modulate the mode of cell death [186, 190].

Many cytotoxic agents induce ROS, including peroxide and O_2_^•−^, which are involved in the induction of apoptotic cell death [201]. H_2_O_2_ can cause the release of cytochrome *c* from mitochondria into the cytosol and H_2_O_2_ may also activate nuclear transcription factors, like NF-*κ*B, AP-1, and p53 [202], which may upregulate death proteins or produce inhibitors of survival proteins. One model proposed for H_2_O_2_ induction of apoptosis is upregulation of the Fas-FasL system, leading to activation of caspase-8 and downstream caspases [203, 204]. It is also possible that NO^•^ may also inactivate several antioxidant enzymes, including catalase, glutathione peroxidase, and superoxide dismutases [205, 206]. Also, NO^•^ has been reported to induce apoptosis by increasing ceramide generation through caspase-3 activation, induction of mitochondrial permeability transition, and activation of the Fas system [207]. 

Certain anti-apoptotic proteins have also been reported to have antioxidant roles. An early suggestion regarding the mechanism of action of Bcl-2 was that it inhibited cell death by reducing the generation of reactive oxidants, thus preventing critical intracellular oxidations that are requisite for the completion of the apoptotic program [208]. However, it is now understood that the reduction in ROS observed with Bcl-2 overexpression is probably the result of its ability to prevent loss of cytochrome *c *from mitochondria. Yet it is interesting to note that separate studies illustrate that Bcl-2-overexpressing cells have higher levels of total cellular GSH [209]. The product of the baculovirus p35 gene, a potent anti-apoptotic protein, is thought to have antioxidant role and is protective against many apoptotic stimuli including growth factor withdrawal, staurosporine, glucocorticoid, and actinomycin-D treatment, and is a broad-spectrum caspase inhibitor [210]. However, caspase inhibition may not be p35's sole mechanism of cytoprotection. Expression of the p35 gene inhibits H_2_O_2_-induced apoptosis in insect cells and may be acting as a sink for free radicals [211]. 

However, ROS are also reported to interfere with the apoptosis death program, compelling cells to adopt an alternative mode of cell death. Apoptotic cell death can be switched to necrosis during oxidative stress by two possible mechanisms: inactivation of caspases or a drop in cellular levels of ATP levels. Caspases contain an active site cysteine nucleophile [212] which is prone to oxidation or thiol alkylation as well as S-nitrosylation [80, 213, 214]. This leads to their inactivation, switching the mode of cell death to necrosis [80, 214]. NO^•^ may act as a molecular switch to control protein function via reactive thiol groups. For example, NO^•^-mediated inhibition of apoptosis in most cases is due to direct inhibition of caspase activity through S-nitrosylation of the active site cysteine conserved in all caspases although indirect effects on caspases can also be a component of toxicity in certain systems [214]. A switch from apoptosis to necrosis can also occur due to a drop in cellular levels of ATP caused by the failure of mitochondrial energy production by oxidants [215, 216]. As mentioned previously ROS may provide a common link between cellular stress signals and the initiation of autophagy, and ROS accumulation has been reported to result in inactivation of the cysteine protease ATG4, which in turn causes accumulation of the ATG8-phosphoethanolamine precursor that is required for the initiation of autophagosome formation [44]. In most circumstances, the induction of an autophagic response serves as a strategy that should ensure the cell's survival [217]. Under certain conditions, however, it may also bring about cell death, although the molecular determinants that may control the switch from survival to death are still poorly defined. In fact, in response to several anticancer drugs ROS can induce autophagic cell death.

## 4. Switch from Prosurvival Signaling to Cell Death Signaling

While conditions of stress stimulate cells to mount protective responses to counteract the effect of the stress on cellular processes, if the stress remains unresolved, eventual death of the cell ensues. This raises key questions about the molecular mechanisms involved in this switch from prosurvival signaling to prodeath signaling. For example, is there a particular molecule that acts as a molecular switch? How do the duration and severity of the stress contribute to activation of this switch? As described above, in the face of exposure to cell stress, the cell mounts protective responses such as the heat shock response, or the unfolded protein response, in order to relieve the stress and promote survival. However, it is known that if the stress is very severe or if it is prolonged, the cell will die in spite of the activation of prosurvival signaling. 

In the case of the response of cells to heat stress, the induction of Hsps does not occur if the stress is too severe and it has previously been suggested that the induction of thermotolerance, that is, Hsp expression, and of cell death is mutually exclusive events within the same cell [87, 195]. In support of this, we have observed that in a culture exhibiting mixed responses to a stressor, that is, expression of Hsps, induction of apoptosis, and induction of necrosis, the expression of Hsps was mainly observed in the surviving cells [196]. However, a recent report suggests that this may not always be the case, as at least one agent, which induces expression of Hsps through direct activation of HSF1, induces apoptosis rather than being protective [218].

During ER stress, IRE1 may be involved in the switch between the prosurvival UPR and initiation of cell death pathways [219]. Interestingly, the three arms of the UPR are thought to be activated sequentially, with PERK being activated most rapidly, followed by ATF6 and then IRE1. This suggests that time is allowed so that PERK and ATF6 may resolve the stress, and although IRE1 also contributes to the prosurvival UPR, it ultimately terminates it by relieving the translational inhibition by inducing p58^IPK^ [20]. If the stress has been resolved, the cell returns to normal, but if not, then apoptosis is initiated, possibly by IRE1-dependent activation of ASK1 and its downstream target JNK. However, recently it has been shown that attenuation of IRE1 can switch the adaptive UPR to apoptosis and that persistent activation of IRE1 increases cell viability upon ER stress, suggesting that the duration of IRE1 signaling may act as a switch [219].

## 5. Stress Responses in Disease States

It is currently understood that a pathological stress response is a hallmark of many common human diseases for a number of reasons. Firstly, the stress stimulus may be too strong and/or prolonged, thereby allowing insufficient time for recovery to the normal status. Secondly, a cell's ability to handle even physiological levels of stress may be altered in disease states, similarly resulting in detrimental outcomes. In the following section, we will provide some selected examples of how pathological handling of stress is one of the major underlying causes of the pathophysiological state in very different types of human diseases.

### 5.1. Diabetes

Loss of function or death of the pancreatic *β*-cells in the Islets of Langerhans in the pancreas is the major pathological feature of diabetes mellitus. The pancreatic *β*-cells have a highly developed secretory system, in which the ER has an integral role, enabling a rapid response to glucose stimulation by producing and releasing large amounts of insulin. Both oxidative stress and ER stress are involved in the failure of pancreatic *β*-cells and development of diabetes. 

The reactive species which play an important role in the pathogenesis of pancreatic *β*-cell loss in diabetes are generated intracellularly when the *β*-cells are targeted by proinflammatory cytokines in autoimmune Type 1 diabetes or when exposed to a hyperglycaemic and hyperlipidaemic milieu in Type 2 diabetes. There is evidence for the participation of both NO^•^ and ROS in the pathogenesis of *β*-cell death in Type 1 diabetes, whereas, for *β*-cell dysfunction in Type 2 diabetes, ROS are the main culprits.

Proinflammatory cytokines, including IL-1*β* (interleukin 1*β*), TNF*α* (tumor necrosis factor *α*), and IFN*γ* (interferon *γ*), released from immune cells infiltrating the pancreas in Type 1 diabetes, target the *β*-cells via their respective receptors [220]. They activate a multitude of signaling cascades, culminating in apoptosis of *β*-cells [221]. A number of steps in this chain of events affect the rate of generation of NO^•^ and ROS. It is evident from studies in patients with diabetes and in animal models of Type 1 diabetes, that IL-1*β* is the key proinflammatory cytokine which significantly contributes to *β*-cell dysfunction and apoptosis in the pathogenesis of Type 1 diabetes. It does so through activation of the transcription factor NF-*κ*B which is responsible for the induction of iNOS and subsequent production of NO^•^ [155, 221]. The production and release of IFN*γ* acts synergistically with IL-1*β*. High concentrations of IFN*γ* are required to potentiate the effects of IL-1*β* on iNOS and NO^•^ production [222]. NO^•^ and ROS seem to also cross-talk with ER stress and UPR [223]. 

IL-1*β* also induces MnSOD (a manganese-dependent SOD isoenzyme) and this results in an increased rate of conversion of O_2_^•−^ into H_2_O_2_ in the mitochondria [224]. Cu/ZnSOD, the cytoplasmic isoenzyme, is unaffected by IL-1*β*. The profile of the effects of TNF*α* and IFN*γ* alone, or in combination with IL-1*β*, on MnSOD is comparable with that of their regulation of iNOS. The effects on the generation of both radicals are not only important in themselves but also affect the balance between NO^•^ and O_2_^•−^, and this can have significant effects on *β*-cell toxicity. A decrease in O_2_^•−^ through MnSOD may present as a protective signal through a reduction of NF-**κ**B activation and other components of the IL-1*β* signaling pathway [225]. On the other hand, an increased conversion rate of O_2_^•−^ into H_2_O_2_ by SOD is likely to increase toxicity to the *β*-cell with its poor enzymatic capacity for H_2_O_2_ inactivation [226, 227].

Another major proinflammatory cytokine, TNF*α*, is released from the infiltrating immune cells speeds up *β*-cell loss significantly, resulting in an accelerated progression of the disease with rapid loss of the entire pancreatic *β*-cell population and Islet mass. Ceramide is likely to play a significant role as a mediator of O_2_^•−^ formation in TNF*α*-mediated toxicity [228], thereby explaining the dominance of ROS in the case of TNF*α* when compared with IL-1*β*. Thus, with a significant contribution of TNF*α* produced by the infiltrating immune cells in Type 1 diabetes the resulting greater cytotoxicity is the result of the more pronounced ROS component of TNF*α* toxicity. 

That the ROS-mediated component of cytokine toxicity primarily targets the mitochondria is shown by the fact that exposure of insulin-producing cells to IL-1*β*, or to a cytokine mixture containing both IFN*γ* and TNF*α*, causes mitochondrial damage, while other subcellular structures remain intact. This damage can be prevented by expression of high levels of catalase or GSH in the mitochondria, but not in the cytosol [228]. IL-1*β* toxicity, mediated through NO^•^ and potentiated by IFN*γ* and TNF*α*, is likely to focus its effects in the cytoplasm. This component will presumably contribute to ER stress, which plays a significant role in dysfunction of *β*-cells under cytokine attack [229].

*β*-Cell loss in Type 2 diabetes is slower than in Type 1 diabetes, typically with a long phase of *β*-cell dysfunction, characterized by defective insulin secretion in response to glucose. In Type 2 diabetes, glucolipotoxicity, rather than proinflammatory cytokines, is considered to be an important contributing factor to *β*-cell dysfunction [230–234]. It is evident from studies on *β*-cells exposed to a combination of high glucose and a saturated fatty acid that NO^•^ generation through iNOS induction does not contribute to *β*-cell dysfunction [235]. 

Increased mitochondrial metabolic flux is required in the *β*-cell for generation of the ATP signal for glucose-induced insulin secretion [236] and its potentiation through fatty acids [231]. On the other hand, increased metabolic flux through the respiratory chain at high glucose and lipid concentrations should increase O_2_^•−^ formation, thereby reducing the mitochondrial membrane potential via uncoupling protein 2 [230, 237]. This should decrease metabolic flux through the respiratory chain and thus reduce O_2_^•−^ production, thereby acting in a protective manner against ROS-induced damage, but, at the same time, attenuating nutrient-induced insulin secretion. This casts doubt on the concept that increased intra-mitochondrial generation of ROS crucially contributes to *β*-cell damage in Type 2 diabetes.

This interpretation is supported by the results of morphological analyses showing that insulin-producing cells exposed to the fatty acid palmitate show no signs of mitochondrial damage, but very pronounced defects of the ER [238], confirming observations of increased ER stress in response to glucolipotoxicity [235]. Thus one of the prominent targets of this free-radical-mediated toxicity might indeed be the ER.

Defects in PERK-eIF2*α* pathways cause Wolcott-Rallison syndrome, a rare infantile-onset insulin-requiring diabetes [239] and PERK-null mice developed similar phenotypes [240]. Mice with mutated proinsulin (proinsulin-2) that cannot form a disulfide bond (Akita mice) also develop severe diabetes which is associated with the ER stress-induced cell death in pancreatic *β*-cells [241, 242]. Mice deficient for p58^IPK^, which suppresses PERK-mediated phosphorylation of eIF2*α*, exhibit apoptosis of pancreatic *β*-cells and diabetes [243]. This suggests that the tight regulation of PERK-eIF2*α* pathway is required for the maintenance of pancreatic *β*-cells. In contrast, some single nucleotide polymorphisms (SNPs) in the ORP150/GRP170 genome of Pima Indians are associated with insulin sensitivity in peripheral tissues [244]. Accordingly, overexpression of ORP150 enhances insulin sensitivity and suppresses oxidative stress but does not improve insulin secretion [245]. These findings suggest that proper functioning of the ER is important for both insulin synthesis in pancreatic *β*-cells and insulin sensitivity in peripheral tissues. Consistent with this hypothesis, chemical chaperones such as 4-phenylbutryic acid and tauroursodeoxycholic acid improved both insulin resistance and insulin synthesis [171, 246].

### 5.2. Parkinson's Disease

Neurodegenerative diseases are characterized by the loss of subsets of neurons. The course of these diseases can last decades, with the accumulation of neuronal loss causing progressively worse symptoms. Postmortem tissue is usually obtained from end-stage patients, at which time many of the evidences regarding the events preceding cell death are long gone. However, there is substantial and growing evidence for the activation of stress responses in neurons in all of the common neurodegenerative diseases. This suggests that when neurons are exposed to stress, they counteract with activation of one or more protective stress responses; however, eventually the neurons are unable to cope and one-by-one they are lost as the disease progresses. There is a growing recognition that protein misfolding and impairment of protein handling play a key role in neuronal cell death in neurodegenerative diseases [153]. 

As an example of stress responses and stress-induced cell death in neurodegenerative disease, we will describe the evidence pertaining to Parkinson's disease. Parkinson's disease is the second most common neurodegenerative disease, affecting mainly people over 55 years and causing progressively worsening motor impairment. It is characterized pathologically by the degeneration of midbrain dopaminergic neurons in the substantia nigra pars compacta and the presence of proteinaceous intracytoplasmic inclusions (Lewy bodies) within the surviving neurons. 

The molecular mechanisms that initiate dopaminergic neuron loss in Parkinson's disease are not known. Evidence from various sources suggest that environmental toxins, genetic predisposition, and aging are important factors in the onset and progression of the disease [247–249]. Insecticides such as rotenone and the mitochondrial toxin 1-Methyl-4-phenyl-1, 2,3,6-tetrahydropyridine (MPTP) cause dopaminergic neuronal loss in animal models and have been implicated in Parkinson's disease itself [250, 251]. To date, mutations in at least 13 PARK genes have been linked to the pathogenesis of familial Parkinson's disease which include mutations in genes that encode the proteins *α*-synuclein, parkin, PTEN-induced kinase 1 (PINK1), DJ-1, leucine-rich repeat kinase2 (LRRK2), Omi/Htra2, and ubiquitin carboxy-terminal hydrolase L1 (UCHL1) [252]. Of these, *α*-synuclein (along with chaperone proteins and ubiquitin) is a major component of Lewy bodies. Parkin and UCHL1 are linked to the ubiquitin-proteasome system that degrades damaged or misfolded proteins [253]. In addition, several of these genes, including parkin, PINK1, DJ-1, and Omi/Htra2 are linked to the mitochondria and may have roles in mitochondrial function and resistance to oxidative stress [254]. 

Mutations in PARK genes, as well as toxins that specifically target dopaminergic neurons, have been strongly linked to the activation of stress responses in dopaminergic neurons. For example, mitochondrial dysfunction due to mutations in certain PARK genes or to environmental toxins is linked with impairment of mitochondrial complex I which causes oxidative stress in affected cells. It has long been known that oxidative stress is a feature of Parkinson's disease and it is observed in experimental models of Parkinson's disease and in tissues from individuals with sporadic forms of the disease [255]. 

Most of the evidence regarding activation of the heat shock response in Parkinson's disease come from models. Targeted overexpression of *α*-synuclein in mouse substantia nigra causes an increase in the expression of Hsp27, Hsp40, and Hsp70 [256, 257] and elevations in Hsp27 are observed in in vitro models of Parkinson's disease using the neurotoxin 6-hydroxdopamine [196]. Recent findings from Parkinsonian patients have described that DnaJB6 is present in the core of Lewy bodies and is also upregulated in astrocytes [258]. DnaJB6 is one of the Hsp40 chaperones, which stabilizes the interactions of Hsp70s with their substrate proteins. In vitro and in vivo models of Parkinson's disease demonstrate that overexpression of Hsps prevents *α*-synuclein aggregation as well as dopaminergic neuronal cell death due to *α*-synuclein and Parkinson mimetic toxins [216, 237, 239–242]. Interestingly, the inducibility of Hsps decreases with aging, which may contribute to the inability of aged neurons to fully protect themselves from stresses such as protein misfolding, aggregation, and oxidative stress [259]. 

Activation of the UPR has been reported in postmortem brain tissue from patients with Parkinson's disease. Specifically, phosphorylated PERK and phosphorylated eIF2*α* have been detected in dopaminergic neurons in the substantia nigra of Parkinson's disease patients [260]. Phospho-PERK immunoreactivity was colocalized with increased *α*-synuclein immunoreactivity in dopaminergic neurons [260]. Supporting evidences from in vitro models of Parkinson's disease show that 6-hydroxydopamine and 1-methyl-4-phenyl-1,2,3,6-tetrahydropyridine (MPP^+^) (Parkinson mimetic drugs) trigger ER stress in dopaminergic neurons [261, 262]. Furthermore, neuronal cultures from PERK knockout mice display an increased sensitivity to 6-hydroxydopamine [262], while a null mutation in CHOP results in a reduction in 6-hydroxydopamine-induced apoptosis in vivo [263]. However, protection was not observed in the chronic MPTP model, despite robust expression of CHOP [263].

The information from models, the genetic information, as well as analysis of postmortem tissue, when taken together, strongly connects the induction of stress responses with the loss of dopaminergic neurons in Parkinson's disease. It is likely that the induction of stress responses is the neurons attempts at protection, which eventually fail with neuronal cell death being the inevitable outcome. Interestingly, these observations are mirrored in research findings of other common neurodegenerative diseases, including Alzheimer's disease and Huntington's disease, indicating the important role for protein misfolding, aggregation and formation of protein inclusions in these chronic diseases [153].

### 5.3. Myocardial Infarction

Cardiovascular disease (CVD), a group of disorders of the heart and the vasculature, includes high blood pressure, coronary heart disease, congestive heart failure, stroke, and congenital heart defects. Apoptotic cell death is a fundamental process in the morphogenesis of the developing heart [264, 265]. Until recently the classical view was that necrosis was the major mode of cardiomyocyte death during CVD. However, accumulating in vitro and in vivo studies provides compelling evidence that terminally differentiated cardiomyocytes, can and do undergo apoptosis [266]. Apoptosis has important pathophysiological consequences, contributing to the loss and functional abnormalities of the myocardium. Cardiomyocyte apoptosis has been reported in a variety of cardiovascular diseases, including myocardial infarction, end-stage heart failure, arrhythmogenic right ventricular dysplasia, and adriamycin-induced cardiomyopathy [267]. Animal models have been instrumental in establishing the occurrence of cardiomyocyte apoptosis and in the elucidation of the apoptotic mechanisms. Features of myocyte apoptosis were first reported in rabbit and rat heart models of MI or ischemia/reperfusion injury [268, 269]. Since these pioneering studies, apoptosis has been repeatedly observed in the injured human heart [270–274]. Due to its sporadic occurrence and the prompt clearance of apoptotic cells by phagocytosis, apoptosis in diseased tissue is grossly underestimated. 

Oxidative damage mediated by free radicals is a contributing factor to ischemia/reperfusion-induced injury in cardiomyocytes [275–278]. Plasma and pericardial fluid obtained from patients with end stage heart failure have increased levels of thiobarbituric acid reactive substances, a commonly used marker of ROS production [279, 280]. Reperfusion is associated with a burst of ROS generated via the mitochondrial respiratory chain, where partial reduction of ubiquinone forms ubisemiquinone combine with oxygen to form O_2_^•−^ radicals [281]. High levels of ROS can lead to mitochondrial damage and dysfunction [282] and can induce apoptosis in cardiac myocytes [275, 276].

In addition, enhanced levels of the heat shock response and UPR have been demonstrated in animal models of myocardial infarction, and overexpression of either Hsps or GRPs enhanced tolerance against ischemia/reperfusion injury in these models [283, 284]. Although Hsps may work upstream of caspase-3 but downstream of cytochrome *c* release [285], GRPs likely contribute to the maintenance of intracellular Ca^2+^ homeostasis [284]. Similarly, overexpression of sarco (endo) plasmic reticulum Ca^2+-^ATPase (SERCA), which regulates intracellular Ca^2+^ homeostasis, improved postischemic cardiac function and decreased myocardial infarction [286].

### 5.4. Cancer

Since tissue homeostasis is the result of a subtle balance between proliferation on one side and cell death on the other side, changes in the rate of cell death can contribute to either the loss or gain of tissue [22]. For example, too little cell death can contribute to tumor formation and is considered to be one of the hallmarks of human cancers [287, 288]. Some oncogenic mutations block cell death pathways creating a permissive environment for genetic instability and resulting in the accumulation of gene mutations leading to tumor initiation and progression [289]. Also, evasion of cell death promotes resistance to immune-based destruction, facilitates growth factor- or hormone-independent survival, and supports anchorage-independent survival during metastasis [288]. In addition, defects in cell death programs may confer resistance to cytotoxic therapies that are currently used in the clinic for the treatment of cancer such as chemotherapy, irradiation, or immunotherapy, since the response of cancer cells to these treatment approaches is, to a large extent, due to their ability to undergo cell death in response to cytotoxic stimuli [290–292].

In principle, the signaling to apoptosis can be blocked in cancers by loss or defective function of proapoptotic molecules, aberrantly high expression of antiapoptotic proteins, and/or by the relative dominance of cell survival signaling pathways. For example, impaired death receptor expression or function has been reported in a variety of human cancers. Reduced expression of CD95 was found in drug-resistant leukemia or neuroblastoma cells, indicating that intact signaling via CD95 is linked to drug response [293, 294]. CD95 mutations have been detected in both hematological malignancies and various solid tumors [295–300]. It is interesting to note that both agonistic TRAIL receptors, that is, TRAIl-R1 and TRAIL-R2, are located on chromosome 8p, a region that is frequently lost in cancers due to heterozygosity [301, 302]. Further, a larger range of antiapoptotic proteins are reported to be expressed at high levels in malignant versus nonmalignant tissue, including death domain-containing proteins that interfere with activation of caspase-8 at the death receptor level such as cellular FLICE-Inhibitory Protein (cFLIP) and phosphoprotein enriched in diabetes/phosphoprotein enriched in astrocytes-15kDa (PED/PEA-15) [303], anti-apoptotic Bcl-2 family proteins such as Bcl-2, Bcl-X_L_, and Mcl-1 [31] and IAPs, including XIAP, cIAP1, cIAP2, survivin and livin [304]. Alternatively, apoptosis regulators with proapoptotic functions have been reported to be lost, mutated or epigenetically silenced in cancers. Examples include epigenetic loss or homo- or heterozygous genomic deletions of caspase-8 [305], single nucleotide substitution or frameshift mutations of the *bax* gene in mismatch repair-deficient colon cancer or hematopoetic malignancies [306, 307], and deletion or epigenetic silencing of the *bim gene *[308–310]. 

It is also now generally accepted that the majority of tumors, due to poor vascularisation of the tumor mass, experience stressful conditions in the tumor microenvironment, including low oxygen supply, nutrient deprivation, and pH changes. These conditions activate a range of cellular stress-response pathways, including the UPR. Recent studies have shown that the UPR plays an important role in tumorigenesis [311–315]. Activation of at least one branch of the UPR has been reported in a number of cancers and many ER chaperones and UPR target genes show increased expression in human tumor samples. Although activation of the UPR has been reported in a variety of human cancers, the role of UPR in different forms of cancer is not yet fully characterized. 

At present it is unclear how tumor cells adapt to long-term ER stress in vivo—whether the protective elements of the response are enhanced, the destructive components suppressed, or if the compromised apoptotic machinery is sufficient to protect them from UPR-induced apoptosis. Given that the UPR can trigger prosurvival and pro-apoptotic signals, it is important to understand how modulation of the UPR alters the balance between these processes and contributes to carcinogenesis in different cell types. The upregulation of UPR in cancers may be beneficial for the tumor cells by increasing the protein folding capacity and prolonging life.

Moreover, altered redox status can promote tumor initiation and progression by blunting cell death pathways. For example, a pro-oxidant intracellular milieu has been linked to carcinogenesis and tumor promotion. To this end, increased signaling via the PI3K/Akt pathway has been shown to result in enhanced intracellular ROS generation [316]. Similarly, cancer cells that constitutively express oncogenic Ras have been reported to harbor higher intracellular levels of O_2_^•−^ and to be resistant to drug-induced apoptosis [317]. 

Hsps, including Hsp90, Hsp70, and Hsp27, are expressed at increased levels in many solid tumors and haematological malignancies. Since various oncogenic proteins that are critically required for the malignant transformation of cells, for example, Ras, Akt, and HER2, are client proteins of Hsp90, elevated levels of Hsp90 favor tumor initiation and promotion [318]. Similarly, the expression of Hsp27 and Hsp70 is abnormally high in cancers [319]. These chaperones participate in carcinogenesis and in cell death resistance by blocking key effector molecules of the apoptotic machinery at the pre- and post-mitochondrial level [319]. Thus, targeting Hsps, for example, with chemical inhibitors, is currently under investigation as anticancer strategy [318].

Error-prone repair or complete failure to repair DNA damage as well as inherited or acquired defects in maintenance systems of the mammalian genome can lead to mutations [185]. In addition, such deficiencies in the DNA damage response contribute substantially to carcinogenesis and promote the progression and treatment resistance of cancer [185].

## 6. Summary and Future Perspectives

Cellular stress responses are an integral part of normal physiology to either ensure the cell's survival or alternatively to eliminate damaged or unwanted cells. Several distinct stress responses can be distinguished, among them the heat shock, unfolded protein, DNA damage, and oxidative stress responses. Despite individual signaling components, these different stress responses can eventually fuel into common cell death effector mechanisms, if the cell is unable to cope with the stress. Whether or not cellular stress triggers cell death or cell survival programs is determined by a set of different factors, among them the initial stress stimulus, cell type, and environmental factors. Because aberrant cellular stress responses are tightly linked to many common human diseases, a better understanding of the underlying molecular mechanisms is expected to enable us to interfere with these processes, for example, to switch such response from cell death into survival programs or vice versa, depending on the desired outcome. In addition, new insights into the mechanistic basis of stress responses will open new perspectives for the development of molecular targeted treatment approaches and thus have a great potential for drug discovery.
